# Reduced frontotemporal connectivity during a verbal fluency task in patients with anxiety, sleep, and major depressive disorders

**DOI:** 10.3389/fneur.2025.1542346

**Published:** 2025-01-30

**Authors:** Fanxi Ding, Yiyang Ying, Yuqing Jin, Xuanru Guo, You Xu, Zhenghe Yu, Haiteng Jiang

**Affiliations:** ^1^Affiliated Mental Health Center & Hangzhou Seventh People’s Hospital, School of Brain Science and Brain Medicine, and Liangzhu Laboratory, Zhejiang University School of Medicine, Hangzhou, China; ^2^The First School of Clinical Medicine, Zhejiang Chinese Medical University, Hangzhou, China; ^3^Department of Psychology and Behavioural Sciences, Zhejiang University, Hangzhou, China; ^4^MOE Frontier Science Center for Brain Science and Brain-Machine Integration, State Key Laboratory of Brain-Machine Intelligence, Zhejiang University, Hangzhou, China; ^5^NHC and CAMS Key Laboratory of Medical Neurobiology, Zhejiang University, Hangzhou, China

**Keywords:** fNIRS, executive function, verbal fluency task, functional connectivity, network-based statistic

## Abstract

**Background:**

It has been well established that psychiatric disorders are often accompanied by cognitive dysfunction. Previous studies have investigated the verbal fluency task (VFT) for detecting executive function impairment in different psychiatric disorders, but the sensitivity and specificity of this task in different psychiatric disorders have not been explored. Furthermore, clarifying the mechanisms underlying variations in executive function impairments across multiple psychiatric disorders will enhance our comprehension of brain activity alternations among these disorders. Therefore, this study combined the VFT and the functional near-infrared spectroscopy (fNIRS) to investigate the neural mechanisms underlying the impairment of executive function across psychiatric disorders including anxiety disorder (AD), sleep disorder (SD) and major depressive disorder (MDD).

**Methods:**

Two hundred and eight participants were enrolled including 52 AD, 52 SD, 52 MDD and 52 healthy controls (HCs). All participants completed the VFT while being monitored using fNIRS to measure changes in brain oxygenated hemoglobin (Oxy-Hb).

**Results:**

Our results demonstrated that MDD, AD and SD exhibited decreased overall connectivity strength, as well as reduced connected networks involving the frontal and temporal regions during the VFT comparing to HC. Furthermore, the MDD group showed a reduction in connected networks, specifically in the left superior temporal gyrus and precentral gyrus, compared to the AD group.

**Conclusion:**

Our study offers neural evidence that the VFT combined with fNIRS could effectively detect executive function impairment in different psychiatric disorders.

## 1 Introduction

Psychiatric disorders, such as major depressive disorder (MDD), anxiety disorder (AD), and sleep disorder (SD), are highly prevalent and have a significant impact on daily function and quality of life (1–3). These three psychiatric disorders are often co-occurred and accompanied by cognitive dysfunction (4). By investigating MDD/AD/SD together, it will shed new insight on the shared and distinguished disease mechanisms, leading to more effective treatment. Moreover, growing evidence suggests that structural and functional abnormalities in the prefrontal cortex (PFC), which is closely related to cognitive function, and its connected regions are characteristic of various psychiatric disorders (5–8). For example, failure to inhibit the posterior cingulate cortex (PCC) and ventromedial PFC during a task in MDD patients were significantly associated with depression severity and hopelessness (9). Decreased cortical activity in the left frontal eye field (lFEF) and right dorsolateral prefrontal cortex (rDLPFC) could be neural markers for anxiety symptoms after controlling depressive symptoms (10). Besides, reduced gray matter volumes (GMVs) in the right superior frontal area (SFG) and left supplementary motor area (SMA) have been observed in SD (11).

Neuropsychological measurements combined with brain imaging techniques can provide a real-time perspective on the state of brain function during cognitive process. fNIRS is a non-invasive neuroimaging optical technique used to measure the concentrations of oxyhemoglobin and deoxyhemoglobin (O_2_Hb and HHb, respectively) changes induced by cognitive stimulation (12–14). Owing to its low cost and ease of application in an ecologically valid setting, fNIRS is gaining popularity in the field of psychiatry (5, 15). For example, fNIRS measurements during emotion- or cognition-related tasks have been suggested as an adjunct test to support the diagnosis of MDD (16–18). Additionally, it has been shown that fNIRS could be used as a measure of the severity of psychotic symptoms (19).

There are many tasks that can be used to detect cognitive impairment in patients with psychiatric disorders. The VFT is a commonly used in neuropsychological test (20), which consists in generating as many nouns as possible that start with a given letter or belong to a semantic category. The VFT relies on executive processes, requiring the retrieval of items from long-term memory storage, the retention of generated words (working memory), the maintenance of cognitive effort, and the inhibition of inappropriate responses. Moreover, the low sensitivity to speech-related motion artifacts and the possibility of replicating VFT commonly used in traditional neuropsychological assessments in natural experimental settings make fNIRS a more suitable method for the study of speech production (21). The combination of fNIRS and VFT has been used in psychiatric research for many years (15, 22–24). On the whole, the common finding is that many psychiatric disorders, including MDD and generalized anxiety disorder (GAD), are related to reduced activity in the frontal or temporal regions during VFT (5, 25–32). For instance, Akiyama et al. (25) found that MDD showed significant deactivation in bilateral fronto-temporal regions compared with controls although there was no significant difference in VFT performance. Besides, a previous study demonstrated that during VFT, the GAD group showed significantly reduced activation of the medial PFC and left ventrolateral PFC compared with the HC group (30).

**Figure 1:**
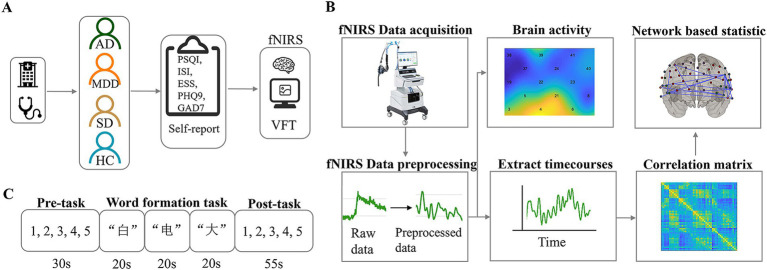
Experimental procedure, task illustrations and data analysis. **(A)** Diagrammatic representation of the experimental procedure. **(B)** Overview of fNIRS data acquisition and analytical pipeline. **(C)** The verbal fluency task protocol.

However, it is still unclear what changes occur in executive function mechanisms among different psychiatric groups with the same cognitive dysfunction. To bridge this gap, our study utilized fNIRS and VFT to examine the neural mechanisms through which these three psychiatric disorders contribute to impairment in individual executive function (Figure 1).

## 2 Materials and methods

### 2.1 Participants

To estimate the proper sample size, we did the calculation using the G*Power software. To achieve a statistical power of 0.85, 204 subjects were needed at least. Therefore, 208 subjects (4 groups, 52 subjects for each group) were enrolled in our study. Fifty-two healthy controls (HCs) were recruited through advertisements, all of whom were required to have intact social functioning and full behavioral and legal responsibility. Participants with the following criterions were excluded: (1) history of neurological or psychiatric illness; (2) family history of psychiatric disorders within two generations; (3) organic brain diseases or severe physical illnesses; (4) female participants who had been in the perinatal period within the past year. The same number of MDD, SD and AD patients matched with HCs in terms of age and gender were recruited from the Hangzhou Seventh People’s Hospital. All patients, recruited from both outpatient and inpatient settings at Hangzhou Seventh People’s Hospital between March 17, 2021, and September 4, 2022, were diagnosed by certified physicians in accordance with the DSM-5 criteria established by the American Psychiatric Association. The demographic information and scale assessment were shown in Table 1. The Chinese version of the Pittsburgh Sleep Quality Index (PSQI) (33, 34) was used to evaluate the patient’s sleep quality in the past month, including seven factors: sleep quality, sleep latency, sleep duration, habitual sleep efficiency, sleep disturbance, use of sleeping medications, and daytime dysfunction. The Chinese version of the Patient Health Questionnaire (PHQ-9) (35, 36) was used to measure the severity of depression in the subjects. The Chinese version of the Generalized Anxiety Disorders Scale (GAD-7) (37, 38) was utilized to screen for generalized anxiety and to assess the severity of its symptoms. The Chinese version of the Epworth Sleepiness Scale (ESS) (39, 40) was used to assess excessive daytime sleepiness, with a total score of 24 points. The Chinese version of the Insomnia Severity Index (ISI) (41, 42) was employed as a tool for insomnia screening, assessing the nature and severity of sleep disturbances in participants. For all scales, higher total scores indicate more severe symptoms.

**Table 1:** Demographic and scale data (M ± SD).

	AD	MDD	SD	HC	*p*	*Post hoc* test
Demographics						
Age (year)	35.98 ± 7.32	35.83 ± 13.43	35.36 ± 10.32	35.92 ± 6.99	n.s.	NA
Gender (F/M)	34/18	38/14	35/17	38/14	n.s.	NA
Scale data						
PSQI	13.59 ± 3.96	3.95 ± 3.27	13.53 ± 3.32	14.76 ± 3.81	<0.001	HC < AD HC < MDD HC < SD
ISI	15.46 ± 6.31	2.33 ± 2.99	14.00 ± 5.56	17.44 ± 5.62	<0.001	HC < AD HC < MDD HC < SD MDD < SD
ESS	5.24 ± 4.60	6.05 ± 3.15	4.23 ± 3.21	7.65 ± 5.29	<0.01	MDD < SD
PHQ9	9.83 ± 4.19	3.45 ± 3.36	6.85 ± 4.00	18.33 ± 5.48	<0.001	HC < MDD < AD <SD
GAD7	9.65 ± 4.57	1.85 ± 2.50	5.54 ± 3.49	14.54 ± 5.13	<0.001	HC < MDD < AD <SD

n.s., not significant at *p* = 0.05 level.

The study procedure was conducted in accordance with the Declaration of Helsinki and approved by the ethics committee of local hospital. All participants provided their written informed consent prior to the study.

### 2.2 Verbal fluency task

The VFT consists of three parts: pre-task rest, word formation task, and post-task rest. The pre-task rest phase lasts 30 s, with the voice prompt “Please repeat the numbers 12345,” and the subject repeats “1, 2, 3, 4, 5” according to the prompt. The task word formation phase lasts 60 s, including a three-character word formation task, such as “白”(white), “电”(electricity), and “大”(big). When the Chinese character prompt appears, the participant needs to try his best to form a word with the specified Chinese characters and say it out. The word formation task and the order of Chinese characters are the same for all subjects. The post-task rest phase lasts 55 s, with the voice prompt “Please repeat the numbers 12345,” and the subject repeats “1, 2, 3, 4, 5” according to the prompt. The subject sits in a chair with his back to the screen in a natural and comfortable posture, and tries to maintain the posture throughout the process.

### 2.3 NIRS measurement

fNIRS data was collected by a multi-channel functional near-infrared spectroscopy imaging system (NirScan-6000C, Huichuang, China), which was equipped with 15 transmitting probes and 16 receiving probes, with a total of 48 measurement channels, covering the frontal lobe and bilateral temporal lobes. The distance between the signal source and the detector was about 3 cm. The absorption of near-infrared light at three wavelengths (730 nm, 808 nm, and 850 nm) was recorded at a sampling rate of 11 Hz.

### 2.4 Data preprocessing and analysis

The collected near-infrared data were preprocessed using the MATLAB function package NIRS-KIT (43). The time derivative distribution repair (TDDR) method was used for motion correction to remove motion tails such as head movement and eye movement (44). A low-pass filter of 0.1 Hz was used to remove system noise and non-task-related physiological tails such as breathing and heartbeat. The optical data were converted into oxygenated hemoglobin (HbO) and deoxygenated hemoglobin (HbR) data according to the modified Beer–Lambert law. The preprocessed clean signals were subsequently analyzed. The task period conditions were convolved with the standard canonical hemodynamic response function (HRF) in the general linear model (GLM) to form the corresponding regressor. This study focused on changes in oxygenated (Oxy-Hb) concentration, because the Oxy-Hb is the most sensitive indicator of regional cerebral blood flow in fNIRS measurement (45, 46).

Based on the above preprocessed data, the data from the word formation phase of the VFT were selected for subsequent functional connectivity analysis using FC-NIRS.^1^ Pearson’s correlation analysis was performed on the word formation phase of data.

### 2.5 Statistical analysis

The differences in the scores of the four groups in PSQI, PHQ9, GAD7, ESS and ISI were analyzed by the one-way ANOVA using SPSS 25.0 (IBM Corp., NY, United States). The one-way ANOVA was used to analyze the brain activation among the four groups. Type I error due to multiple comparisons across voxels was controlled by false discovery rate. Statistical significance was set at *p* < 0.05. Similarly, one-way ANOVA was used to analyze the differences in functional connectivity among the four groups.

The network-based statistic (NBS) (47, 48) was performed to identify networks of brain regions that showed significant between-group differences in inter-regional functional connectivity. Particularly, a one-way ANOVA was used to test between-group differences in correlation coefficients at each of the (48 × 47)/2 = 1,128 unique region pairings. Pairs of regions with a *F*-statistic (absolute value) exceeding an uncorrected threshold of 3.5 (*p* < 0.001) were systematically searched for any interconnected networks, known in graph theory as connected components, that might serve as evidence of differences between the groups. A familywise error (FWE)-corrected *p*-value was then attributed to each network using permutation testing. For each permutation, participants were randomly swapped between the AD, SD, MDD and HC groups. The NBS was then applied to the randomized data, and the size of the largest network (connected component) was recorded. A total of 5,000 permutations were generated in this way to produce an empirical null distribution for the largest network size. Finally, a corrected *p*-value for a network of size *k* in the original data was calculated by comparing it to the largest network in permuted datasets. This proportion of permutations where the largest network equals or exceeds *k* offers weak control of the FWE (49, 50).

To visualize the results, the GraphPad Prism 8 and BrainNet Viewer (51) were used to generate figures.

## 3 Results

### 3.1 Demographic and clinical characteristics

The demographic characteristics of participants in each group are presented in Table 1. There were no significant differences in terms of age [*F* (3, 204) = 0.039, *p* > 0.05] and gender [*χ*^2^ (3) = 0.075, *p* > 0.05] among four groups. However, PSQI, ISI, ESS, PHQ9 and GAD7 were significantly different (Table 1).

### 3.2 Decreased overall functional connectivity differences in disease groups

**Figure 2:**
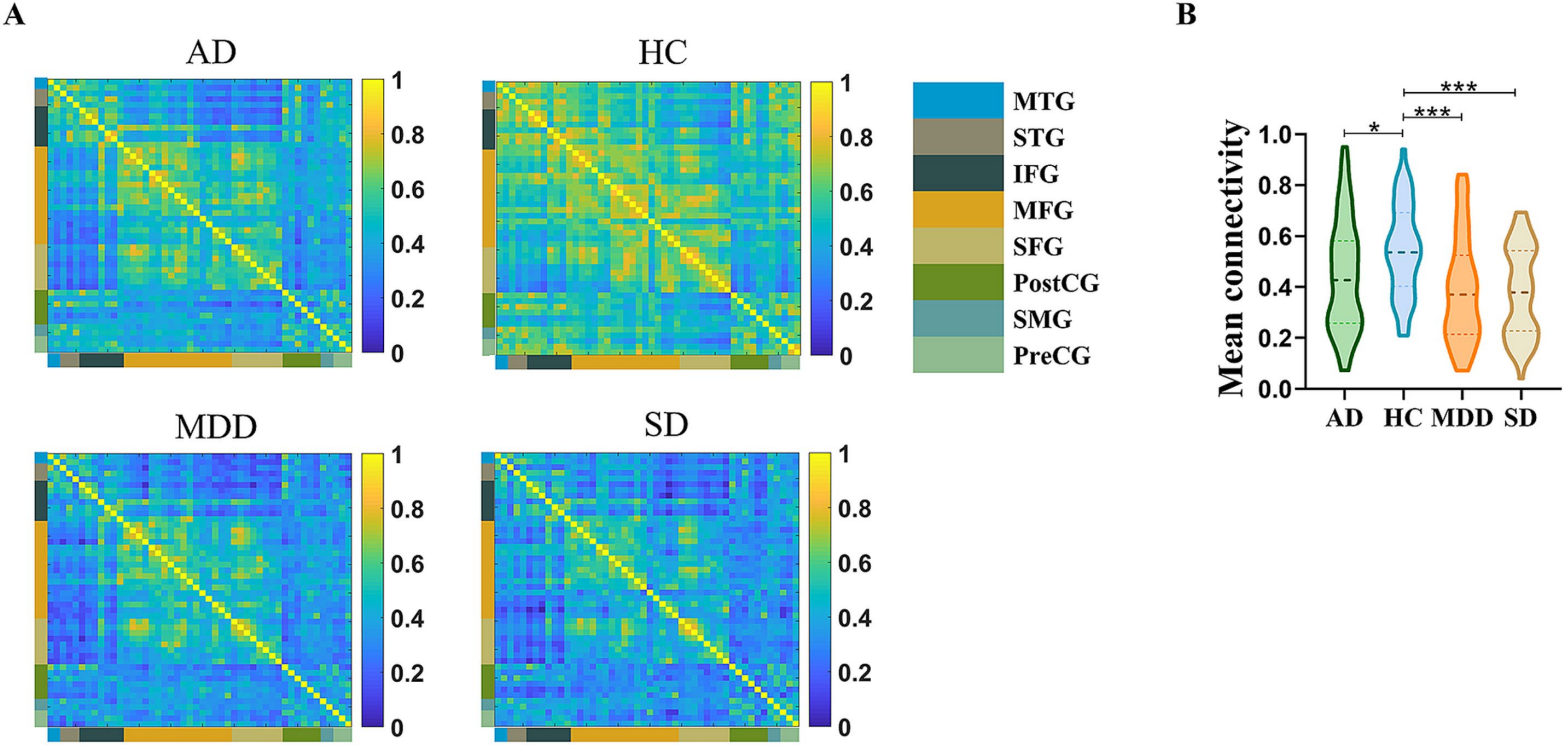
**(A)** Mean channel-channel connectivity of four groups. **(B)** Statistical differences among four groups. The HC group had a significantly higher mean channel-to-channel connectivity strength than the AD, MDD, and SD groups. ^*^p < 0.05 and ^***^p < 0.001.

Prior to the functional connectivity statistic, we compared the brain activations among the four groups during the VFT and no significant differences were identified after multiple comparison correction. After functional connectivity calculation, four 48 × 48 correlation matrices were generated for the MDD, AD, SD and HC groups (Figure 2A). One-way ANOVA showed that the main effect of group was significant [*F* (3, 204) = 17.087, *p* < 0.001, *η*_p_^2^ = 0.201]. *Post hoc* comparisons indicated that the HC group (M = 0.54, SD = 0.18) exhibited significantly higher functional connectivity strength compared to the MDD [M = 0.39, SD = 0.21; *t* (102) = 4.066, *p* < 0.001, Cohen’s *d* = 0.797], AD [M = 0.44, SD = 0.22; *t* (102) = 2.472, *p* < 0.05, Cohen’s *d* = 0.485] and SD [M = 0.38, SD = 0.18; *t* (102) = 4.701, *p* < 0.001, Cohen’s *d* = 0.922] groups (Figure 2B).

### 3.3 Network specific anomalies among different groups

**Figure 3:**
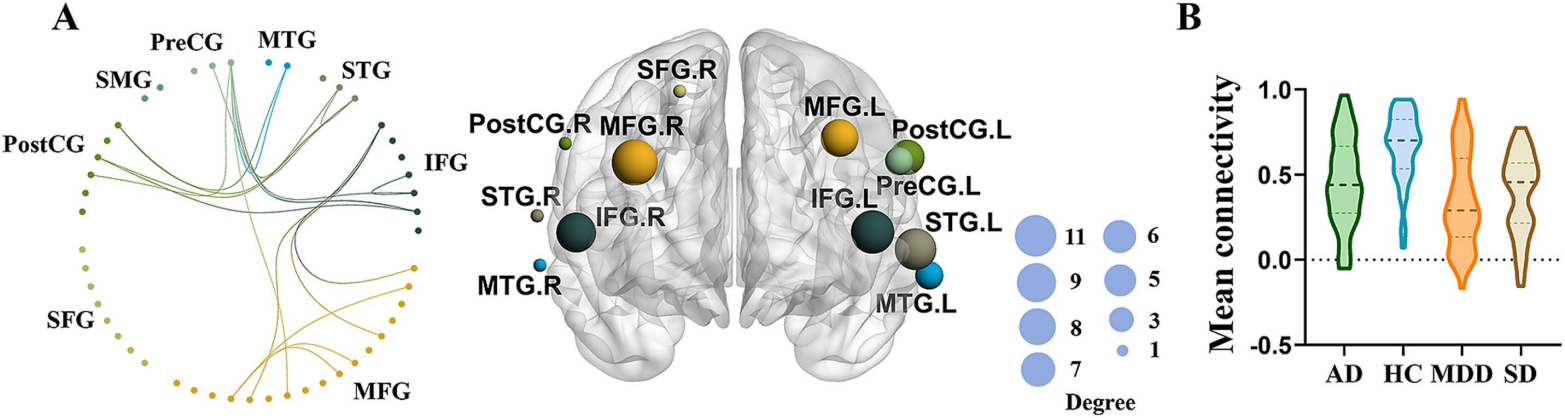
**(A)** Significant network differences among four groups identified by NBS (left panel), while the size of the node on the right panel indicates the number of connections. **(B)** Mean functional connectivity of the significant network in MDD, AD, and SD compared to HC group.

**Figure 4:**
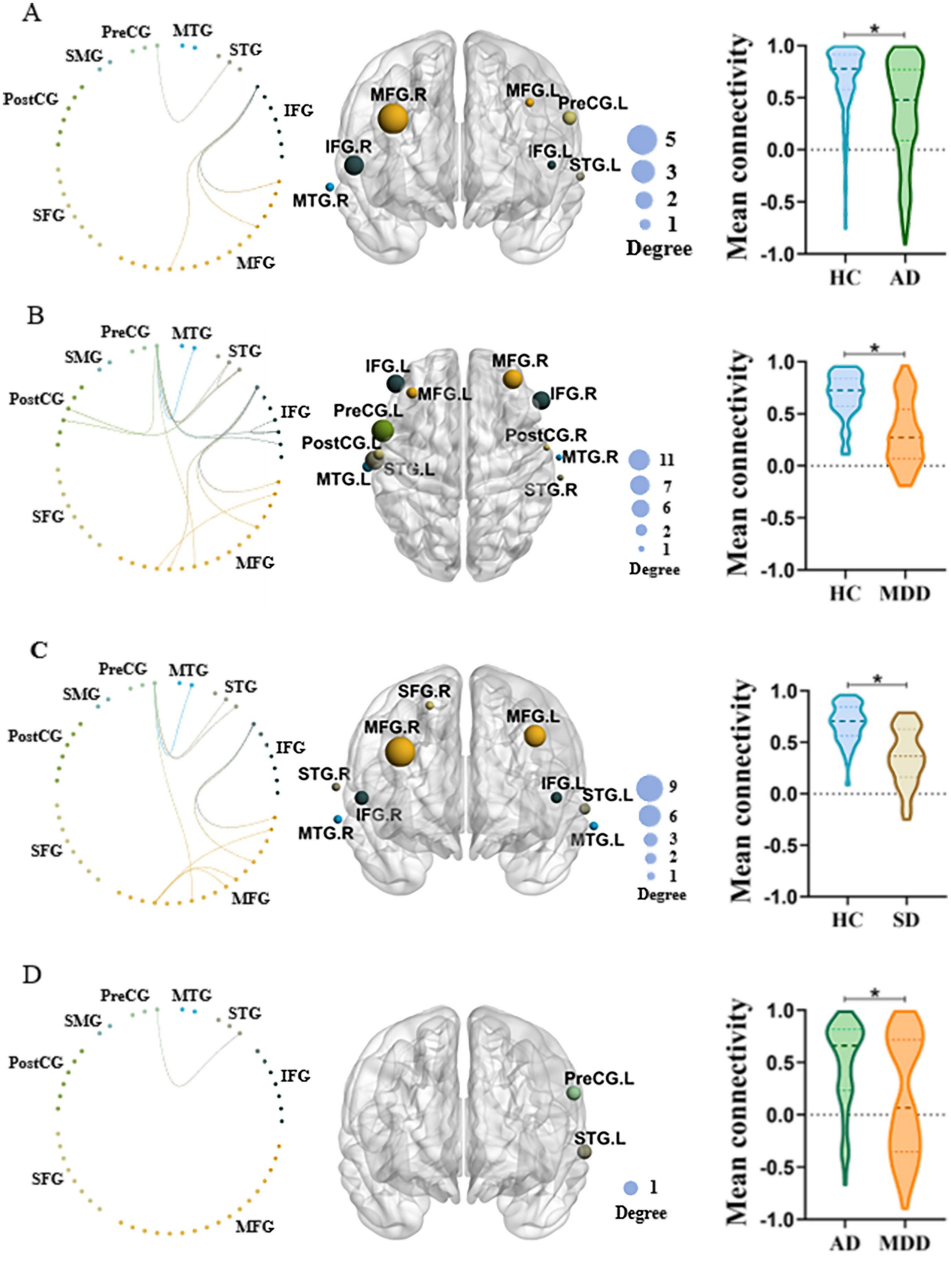
**(A)** NBS comparison between AD and HC. Left panel: Significantly different functional connectivity network between two groups. Middle panel: The most involved regions in the significant network. Right panel: Mean functional connectivity of the significant network in two groups. The size of the node on the middle panel indicates the number of connections. **(B)** Similar to **A** but the comparison between MDD and HC. **(C)** Similar to **A** but the comparison between SD and HC. **(D)** Similar to **A** but the comparison between MDD and AD.

The NBS identified significant differences in network connectivity among AD, SD, MDD and HC groups. A network comprising 36 edges and 26 nodes was significantly different in four groups (*p* < 0.05, FWE-corrected; Figures 3A,B). These networks nodal regions mainly included the bilateral middle temporal gyrus, bilateral superior temporal gyrus, bilateral inferior frontal gyrus, bilateral middle frontal gyrus, bilateral postcentral gyrus, right superior frontal gyrus and left precentral gyrus. When comparing the network between every two groups, four significant networks were identified between AD and HC groups specifically (Figure 4A). The involved nodal regions mainly included the bilateral middle frontal gyrus, bilateral inferior frontal gyrus, right middle temporal gyrus, left superior temporal gyrus and left precentral gyrus. Moreover, a single connected network comprising 20 nodes and 24 edges exhibited significantly reduced connectivity in MDD compared to the HC group (Figure 4B). The affected nodal regions mainly included the bilateral inferior frontal gyrus, bilateral middle frontal gyrus, bilateral middle temporal gyrus, bilateral superior temporal gyrus, bilateral postcentral gyrus and left precentral gyrus.

Besides, a single connected network with 18 nodes and 20 edges exhibited significantly decreased connectivity in SD when comparing to HC (Figure 4C). The involved nodal regions primarily consisted of the bilateral inferior frontal gyrus, bilateral middle frontal gyrus, bilateral superior temporal gyrus, bilateral middle temporal gyrus, right superior frontal gyrus and left precentral gyrus.

Lastly, MDD demonstrated significantly decreased network connectivity than AD (Figure 4D). The network mainly involved nodal regions predominantly comprised the left superior temporal gyrus and the precentral gyrus.

## 4 Discussion

This study found that the overall mean functional connectivity strength was significantly weaker in MDD, AD, and SD patients than the HC group. Specifically, decreased connected networks, particularly in the frontal and temporal regions, were observed in patients compared to HC group. In addition, the MDD group, exhibited decreased connected networks including the left superior temporal gyrus and precentral gyrus comparing to the AD group. Interestingly, no significant differences of brain activation were found among four groups during the VFT revealed by one way ANOVA.

The brain is widely regarded as a dynamic, interconnected network of functions. Numerous neuroimaging studies on patients with psychiatric disorders have focused on the resting-state functional connectivity (RSFC) (52–55). For instance, a previous study revealed that patients with affective disorders exhibited a significantly decreased intra-regional and symmetrically interhemispheric RSFC in the prefrontal cortex when compared to the HC group (55). To our knowledge, limited research on psychiatric disorders has focused on task-related functional connectivity. While RSFC primarily accounts for spontaneous fluctuations in brain activity, task-related functional connectivity provides insights into the brain’s dynamic responses during specific cognitive or behavioral tasks (56, 57). Therefore, a deeper insight into task-related functional connectivity could enhance our understanding of the fundamental aspects of cognitive function in patients with psychiatric disorders (58). The present study found that, compared to the HC group, all patients exhibited significantly weaker channel-to-channel connectivity strength when performing the VFT. Notably, it should be pointed that our results are consistent with most previous studies. For instance, a neuroimaging study revealed decreased channel-to-channel connectivity strength in brain regions mainly located in the prefrontal cortex of patients with chronic insomnia during VFT when compared to HCs (59). Moreover, Dong et al. (60) found that the strength of the prefrontal functional connectivity in patients with MDD was lower than HCs. However, a few studies demonstrated decreased frontal activation during VFT among MDD, AD and SD compared with HCs (25, 30, 61), which were not observed in our study. The inconsistence may be attributed to the statistical differences because we were doing multiple group comparisons rather than two group comparisons. These results suggest that the three psychiatric disorders caused damage to the brain regions related to executive functions.

Furthermore, our study found that patients with AD, SD, MDD showed decreased connected networks, including the frontal and temporal regions, relative to the HC group. Interestingly, these three psychiatric disorders all demonstrated reduced network connectivity comparing to HCs. Specifically, AD showed decreased connected networks including the bilateral middle frontal gyrus, bilateral inferior frontal gyrus, right middle temporal gyrus, left superior temporal gyrus and left precentral gyrus. MDD demonstrated weaker connectivity in networks involving the bilateral inferior frontal gyrus, bilateral middle frontal gyrus, bilateral middle temporal gyrus, bilateral superior temporal gyrus, bilateral postcentral gyrus and left precentral gyrus. In patients with SD, reduced connectivity was observed in networks comprising the bilateral inferior frontal gyrus, bilateral middle frontal gyrus, bilateral superior temporal gyrus, bilateral middle temporal gyrus, right superior frontal gyrus, and left precentral gyrus. Overall, these findings reveal new insights in pathological mechanisms among different psychiatric groups with the same cognitive dysfunction. Neuroimaging studies indicate that verbal fluency depends on the coordinated activity of various brain regions, particularly in the frontal and temporal lobes of the left hemisphere (62). Psychiatric patients often exhibit structural and functional abnormalities in the frontal and temporal lobes (6–8). For example, AD show reduced prefrontal cortex thickness compared to HC (63). Moreover, SD are associated with reduced gray matter volumes (GMVs) in the frontal lobe (11). Previous studies revealed that the frontal lobe damage results in impaired phonemic fluency (64–66). Additionally, the VFT is closely related to the process of retrieval, while temporal and frontal lobes have been suggested to play a critical role in memory retrieval (67, 68). In this study, participants were instructed to form words with specified Chinese characters, involving phonemic verbal fluency. Therefore, the frontal and temporal lobes damages in AD, SD and MDD might lead to decreased frontotemporal network connectivity during the VFT task. Moreover, the MDD group displayed decreased connected networks including the left superior temporal gyrus and precentral gyrus compared to the AD group. This finding may suggest that MDD leads to greater impairment in executive function than AD.

Beyond the three psychiatric disorders discussed in this study, other mental disorders have also been investigated during the VFT with fNIRS. For instance, a previous study showed decreased activation in the left ventrolateral PFC in schizophrenia patients during the VFT (69). Similarly, other studies also found that bipolar disorder and obsessive-compulsive disorder patients exhibited significantly lower frontal and temporal activations during VFT compared to HCs (70, 71). Moreover, brain network analysis under VFT during fNIRS measurement could also be used for the diagnosis of other psychiatric disorders (23, 24). Specifically, a prior study proposed a seed-based FC approach to discern schizophrenia using fronto-temporal Oxy-Hb data during the VFT, with classification performance surpassing that of most methods described in previous studies (24). These evidences showed that the VFT combined with fNIRS is an effective way to support psychiatric disorders diagnosis. While cross-disease diagnosis is critical for precision medicine, most previous studies investigated single type disorder (26, 72). To the best of our knowledge, we are the first to investigate MDD, AD and SD together under VFT task with functional connectivity analysis and discover the common and distinct pathological brain network alternations, which could guide more precise treatment and better outcome.

Our current study had several limitations. First, the number of phrases generated during VFT were not recorded, so the behavior performance could not be analyzed further. Second, in line with previous research, this study did not account for global physiological noise (e.g., skin blood flow). However, recent developments have introduced methods to mitigate the effects of extracranial tissue. Consequently, global physiological noise should be analyzed using these new techniques (e.g., wavelet-based method) (73, 74). Thirdly, the study did not take the prior and ongoing treatments into account, which could potentially impact our findings. Fourth, the scale scores with significant differences between the groups were not controlled as covariates, which may affect the results. Fifth, patients were classified according to their primary diagnosis, which might overlook the impact of comorbid symptoms on the brain activity and need to be investigated further in future.

## 5 Conclusion

In conclusion, our study demonstrated that patients with MDD, AD and SD exhibited decreased connectivity in the frontal and temporal regions during the VFT. Additionally, the MDD group showed decreased connected networks including the left superior temporal gyrus and precentral gyrus, compared to AD group. These findings indicated that the VFT could be an effective tool for detecting executive function impairment in psychiatric disorders.

## Data Availability

The raw data supporting the conclusions of this article will be made available by the authors, without undue reservation.

## Footnotes

^1^ https://www.nitrc.org/projects/fcnirs/
